# Enhanced production of cordycepin in *Ophiocordyceps sinensis* using growth supplements under submerged conditions

**DOI:** 10.1016/j.btre.2020.e00557

**Published:** 2020-11-10

**Authors:** Vikas Kaushik, Amanvir Singh, Aditi Arya, Sangeeta Chahal Sindhu, Anil Sindhu, Ajay Singh

**Affiliations:** a Department of Biotechnology, Deenbandhu Chhotu Ram University of Science and Technology, Murthal, 131039, Sonepat, Haryana, India; b Department of Chemistry, Deenbandhu Chhotu Ram University of Science and Technology, Murthal, 131039, Sonepat, Haryana, India; c Department of Foods and Nutrition, Chaudhary Charan Singh Haryana Agricultural University, Hisar, 125004, Haryana, India; d Haryana Agro Industries Corporation, Research and Development Centre, Murthal, 131039, Sonepat, Haryana, India

**Keywords:** RP-HPLC, Reversed-phase high-performance liquid chromatography, qRT-PCR, Quantitative reverse transcriptase-polymerase chain reaction, mRNA, Messenger ribonucleic acid, cDNA, Complementary deoxyribonucleic acid, dNTP, Deoxyribonucleotide triphosphate, mTOR, Mammalian target of rapamycin, SDA, Sabouraud dextrose agar, MgSO_4_, Magnesium sulfate, KH_2_PO_4_, Potassium dihydrogen phosphate, ANOVA, Analysis of Variance, SEM, Standard error mean, Medicinal mushroom, Cordycepin production, Mycelial biomass, Growth supplements, Cordycepin biosynthesis pathway

## Abstract

Cordycepin is a crucial bioactive compound produced by the fungus *Cordyceps* spp. Its therapeutic potential has been recognized for a wide range of biological properties such as anticancer, anti-diabetic, antidepressant, antioxidant, immunomodulation, *etc.* Moreover, its human random clinical trials depicted a promising anti-inflammatory activity that reduced the airway inflammation remarkably in asthmatic patients. But its overexploitation and low production of cordycepin in naturally growing biomass are insufficient to meet its existing market demand for its therapeutic use. Therefore, strategies for enhancement of cordycepin production in *Cordyceps* spp. are warranted. However, specifically, wild type *Ophiocordyceps sinensis* possesses a very low content of cordycepin and has restricted growth in natural mycelial biomass. To overcome these limitations, this study attempted to enhance cordycepin production in its mycelial biomass *in vitro* under submerged conditions by adding various growth supplements. The effect of these growth supplements was evaluated by reversed-phase high-performance liquid chromatography (RP-HPLC) which demonstrated that among nucleosides- hypoxanthine and adenosine; amino acids-glycine and glutamine; plant hormones- 1-naphthaleneacetic acid (NAA) and 3-indoleacetic acid (IAA); vitamin-thiamine (B_1_) from each group of growth supplements yielded a higher amount of cordycepin with 466.48 ± 3.88, 380.23 ± 1.78, 434.97 ± 2.32, 269.78 ± 2.92, 227.61 ± 2.34, 226.02 ± 1.69 and 185.26 ± 2.35 mg/L respectively as compared to control with 13.66 ± 0.64 mg/L. Further, at the transcriptional level, quantitative real time-polymerase chain reaction (qRT-PCR) analysis of genes associated with metabolism and cordycepin biosynthesis depicted significant upregulation of major downstream genes- *NT5E*, *RNR, purA,* and *ADEK* which corroborated well with RP-HPLC analysis. Taken together, the present study identified growth supplements as potential precursors to activate the cordycepin biosynthesis pathway leading to improved cordycepin production in *O. sinensis*.

## Highlights

• The growth supplements remarkably enhanced the cordycepin production.
• Hypoxanthine and adenosine yielded higher amount of cordycepin.
• The potent growth supplements activated cordycepin biosynthesis pathway.
• Upregulation of *RNR*, *NT5E*, *ADEK* and *purA* mRNA expression was observed.

## 1 Introduction

*O. sinensis* (traditionally known as *Cordyceps sinensis*) is an entomo-pathogenic fungus that belongs to the Ophiocordycipitaceae family classified under the category of medicinal mushrooms [1]. This wild macrofungus exists in different forms such as fruiting bodies and mycelial biomass. Its extract possesses immense medicinal properties due to the presence of bioactive compounds such as cordycepin, cordycepic acid, cordymin, *etc.* [[2], [3], [4], [5], [6], [7]]. Wild *O. sinensis* is a rare and endangered species that is found at high altitudes (3000−5000 m) in the Himalayan range [8]. Due to its limited natural resources, increasing demand for its biomass cannot be fulfilled through naturally available fruiting bodies. Therefore, the present study has attempted to cultivate *O. sinensis* in artificial conditions and enhanced cordycepin production in its biomass. Alternatively, cordycepin could be synthesized by chemical method but the chemical synthesis of cordycepin, as well as its purification is a very complicated and tedious process which requires a large volume of organic solvents to be discharged imposing serious health-related issues to human health and environment. Also, the separation of pure cordycepin compound is hardly achievable rendering with the higher residual of tin with final product suggesting the requirement of improvement in its biosynthesis process [[9], [10], [11]]. Recent reports revealed the production of cordycepin *via* the utilization of liquid surface and/or submerged fermentation technique [[12], [13], [14]]. But in case of *O. sinensis*, it is very difficult to produce fruiting bodies that take several months with a lower amount of bio metabolites [15]. Therefore, mycelial biomass production through fermentation technique is a better alternative to produce bioactive compounds like cordycepin. Several studies have claimed that cordycepin is available from both natural and cultured mycelial biomass and can be easily detected by chromatographic techniques [16]. Though naturally growing fruiting bodies or cultured mycelial biomass contain a very low amount of cordycepin but the cordycepin content in mycelial biomass is highly comparable to its amount in naturally growing fruiting bodies [5]. This led us to make an initial attempt to enhance the cordycepin production in its mycelial biomass grown under artificial conditions as compared to naturally growing wild fruiting bodies.

Literature suggests that several strategies have been devised for its biomass production *in vitro* such as solid and liquid fermentation for mycelial biomass and isolation of bioactive compounds [[17], [18], [19]]. However, submerged fermentation is the most promising method because culture conditions and other parameters can easily be controlled. The optimization of culture conditions for enhanced production of cordycepin by adding growth supplements and additives is easier using submerged fermentation as compared to solid cultivation [17]. Interestingly, artificial mycelial biomass produced through submerged fermentation has similar therapeutic potential and less toxicity as compared to biomass obtained from wild *O. sinensis* [20] paving ways for its enhanced cultivation of at large scale. In terms of cordycepin production, *C. militaris* is considered to be more suitable host than *O. sinensis* due to the presence of higher cordycepin amount in it than *O. sinensis.* But recently Ji et al. [21] suggested that *O. sinensis* have more medicinal effects as compared to *C. militaris*. For instance, *O sinensis* improves the overall intestinal health by decreasing the pH of gut environment and enhancing the growth of beneficial genera including *Phascolarctobacterium* and *Bifidobacterium*, as compared to *C. militaris* [21]. Intriguingly, Chen et al. [22] reported a higher content of unsaturated fatty acids, peptides and mannitol in natural *O. sinensis* as compared with cultured *C. militaris* suggesting *O. sinensis* to be a better nutritional supplement thereby rationalizing the use of *O. sinensis* in the present study [22]. However, to confirm whether *C. militaris* can replace O. sinensis completely needs further investigation.

Cordycepin (3′-deoxyadenosine) is the signature bioactive compound of *Cordyceps* spp. which is considered as nucleoside antibiotic and possesses immense pharmacological activities such as antimicrobial [23,24], antiviral [25], antioxidant [26], antifungal [27], anti-angiogenesis [28], anti-platelet aggregation [58], immunomodulatory [59], anti-pigmentation [60] and anti tumor [29]. It is also known to interfere with different molecular processes including purine biosynthesis [30,31], DNA/RNA synthesis [32], and mTOR signaling transduction pathway [33]. Based on these *in vitro* studies, several *in vivo* preclinical studies have been conducted to evaluate its clinical efficiency in various disorders such as depression [34], steroidogenesis [35], hyperglycemia [36], vascular disorders [37], *etc.* Even, a human random controlled trial of cordycepin in asthmatic patients depicted that it reduced the airway inflammation by lowering the serum levels of IgE, sICAM-1, IL-4, and MMP-9 [38]. Another clinical trial is being carried out as a combinatorial drug with Pentostatin to treat patients with refractory lymphocytic or chronic myelogenous leukemia (ClinicalTrials.gov Identifier: NCT00003005). Although, cordycepin is also reported to possess an anticancer activity but simultaneously, it exhibits some toxicity towards healthy erythrocytes [39] as well as some impairment in healthy organs *in vivo* in mice [61]. This encourages researchers to further determine the optimum and non-toxic dose of this drug candidate as its therapeutic applications cannot be discounted. In addition, this molecule has recently been reported to have herbicidal properties which could potentially replace problematic or toxic herbicide such as glyphosate [40]. These diverse biological activities of cordycepin alone necessitate its large scale production as wild growing fruiting bodies cannot accumulate the required amount of cordycepin. Previous studies reported that cordycepin production in *C. militaris* could be improved by adding different growth supplements such as glycine, adenosine, inosine, thiamine, glutamine and, other growth supplements [[41], [42], [43]]. While there are no reports in the literature for improving cordycepin production in *O. sinensis* using growth supplements. Hence, we hypothesized that growth supplements can either directly or indirectly participate in the cordycepin biosynthesis pathway to enhance cordycepin production in its mycelial biomass. This incited us to grow *O. sinensis* under *in vitro* conditions using submerged fermentation by utilizing different groups of growth supplements. Therefore, to the best of our knowledge, for the first time, this study has shown the effect of different groups of growth supplements on cordycepin production in CS2973 strain isolated from wild growing *O. sinensis.* Often, the synthesis of bioactive compounds is regulated by an array of genes that get upregulated or downregulated during the biosynthesis pathway in the host. Hence, mRNA expression analysis of genes involved in the cordycepin biosynthesis pathway was also performed to determine the effect of growth supplements on potential target genes whose modulation may improve cordycepin production at large scale avoiding any mutational technologies.

## 2 Materials and methods

### 2.1 Collection of wild *O. sinensis*

Fresh fruiting bodies of *O. sinensis* were collected from the Pithoragarh region in Uttarakhand, India. CS2973 strain was isolated from these fruiting bodies and confirmed as *O. sinensis* anamorph both morphologically and through molecular identification, as described previously [44]. Gene sequence information of the isolated strain was deposited at the National Centre for Biotechnology Information (NCBI) under accession no. MG564345. Complete details of isolation and molecular identification of CS2973 strain have been reported in our previous study [44]. Briefly, the CS2973 mycelial culture was grown on a petri plate and inoculum for sub-culturing was prepared by punching out 5 mm potato dextrose agar (PDA) discs using a sterilized cork borer. Further, the discs possessing CS2973 culture was inoculated into 250 mL Erlenmeyer flask with 100 mL of liquid culture medium at 20 °C on a shaking incubator for 10 days. This culture medium was used as inoculum for further expanding the culture [44].

### 2.2 Submerged culturing of CS2973

The optimal liquid culture medium for culturing of CS2973 was composed of 20 g/L sucrose, 10 g/L corn steep powder, 1 g/L MgSO_4_, 0.1 g/l KH_2_PO_4._ The optimal culture conditions for cordycepin production from mycelial biomass were: temperature-20 °C; pH-6; incubation time-10 days and agitation speed-150 rev min^−1^. Initially, starter culture was prepared by inoculating liquid media with a 1 cm piece of solid mycelium grown on sabouraud dextrose agar (SDA) medium. The liquid culturing of CS2973 was performed in a 250 mL shaking flask filled with 100 mL culture media inoculated with 5% inoculum from starter culture. The mycelial biomass from these culture flasks was harvested by filtration through Whatman filter paper which was further dried in a hot air oven at 45 °C and ground to powder.

### 2.3 Supplementation of growth supplements for enhancement in cordycepin production

To increase the cordycepin production in CS2973, four categories of growth supplements were selected and added to the liquid basal culture medium at a working concentration based on literature [42]. The stock solution of each growth supplement was prepared and a working solution was added after filter sterilization through 0.22 μm syringe filters. A list of growth supplements used for enhancement of cordycepin production is given in Table 1.

**Table 1:** Different categories of growth supplements for enhancement of cordycepin production in CS2973.

**Growth supplements**	
**(A) Amino acids-1 g/L**	**(B) Nucleosides-100 mg/L**
Glycine	Hypoxanthine
Glutamine	Adenosine
Glutamic acid	Adenine
Aspartic acid	Thymidine
Aspargine	Thymine
	Inosine
	Uridine
**(C) Plant growth hormones-100 mg/L**	**(D) Vitamins-100 mg/L**
NAA (1-naphthaleneacetic acid)	B_1_(thiamine)
IAA (3-indoleacetic acid)	B_6_ (pyridoxine)
2,4- D (2,4-dichlorophenoxyacetic acid	B_7_ (biotin)
BAP (benzyl amino purine)	C (ascorbic acid)

### 2.4 Quantification of cordycepin content in mycelial biomass

Cordycepin content in the mycelial biomass of CS2973 was analyzed by RP-HPLC (reversed-phase high-performance liquid chromatography) technique following a reported procedure with slight changes [45]. Briefly, to negate the effect of improved biomass on cordycepin production, equal amount i.e 1 *g* of dried CS2973 mycelial powder was dissolved in 20 mL of 70 % methanol in distilled water for each treated group for 1 h under sonication to prepare the mycelial extract. This extract was then subjected to centrifugation at 4000 x g for 30 min followed by its filteration using a syringe filter (0.22 μm) prior to injection into RP-HPLC system [45]. The mobile phase consisted of methanol/ultrapure water (10:90) with 10 mmol/L KH_2_PO_4_ dissolved in it. The column was set at 40 °C and the flow rate was maintained at 1.0 mL/min to carry out elution. The RP-HPLC system was equipped with a UV detector and the chromatogram was analyzed by recording the absorbance at 261 nm. The standard calibration curve was plotted using standard cordycepin at different concentrations (50, 100, 150, 200, and 250 μg/mL) and used to estimate the cordycepin content in various treated groups of mycelial biomass.

### 2.5 RNA isolation

RNA isolation was performed using the total RNA isolation kit (Promega Corporation, Wisconsin, USA). In this method, 100 mg of CS2973 mycelia were homogenized in liquid nitrogen followed by the addition of 250 μL RNA lysis buffer and the lysate was vortexed for 30 s. Next, 350 μL of RNA dilution buffer was added and incubated for 5 min at room temperature (RT) followed by centrifugation at 10,000 rev min^−1^ for 15 min at 4 °C. The aqueous phase present in the upper layer was collected in fresh tube and 200 μL of ethanol was added for RNA precipitation. Further, RNA was eluted following the manufacturer’s protocol The purified RNA was quantified and stored at -80 °C for further use.

### 2.6 cDNA synthesis

1μg of RNA was used as a template for cDNA synthesis using cDNA synthesis kit (Promega Corporation, Wisconsin, USA) according to the manufacturer’s protocol.After completion of the reaction, cDNA was stored at −20 °C which was further used for qRT-PCR analysis.

### 2.7 Quantitative mRNA expression analysis of genes involved in cordycepin biosynthetic pathway

50 ng of cDNA was added to the SYBR green real-time master mix (2X) per reaction using 1 pM of each primer specific for genes involved in the biosynthesis of cordycepin. Real-time amplification or expression of each gene for each growth supplement was performed with a Bio-Rad real-time PCR machine (CFX96) at standardized annealing temperature according to the manufacturer’s protocol. qRT-PCR (quantitative real-time polymerase chain reaction) analysis was used to perform mRNA expression analysis of genes involved in cordycepin biosynthesis- PFK, Phosphofructokinase; SAICAR synthetase; AMPD, AMP deaminase; ADEK, Adenylate kinase; NT5E, 5′-Nucleotidase; RNR, Ribonucleotide reductase; purA, Adenylosuccinate synthase; purB, Adenylosuccinate lyase; purC, Phosphoribosyl-aminoimidazole-succinocarboxamide synthase; purl, Phosphoribosyl-formyl-glycinamidine synthase; PRPS, Ribose-phosphate pyrophosphokinase 1 [2,46]. The primer sequence information of these genes along with accession number is provided in Table 2. Each growth supplement was screened for expression of these genes involved in the biosynthetic pathway of cordycepin. The Ct (Threshold cycle) values of gene expression were normalized to the eukaryotic housekeeping gene, 18S ribosomal RNA, and values represented in the graph are the relative fold change of untreated sample as a control which was calculated using a standard delta-delta Ct method:

Relative fold change = 2^ (-ΔΔCt)

Where ΔΔCt= (ΔCt of treated - ΔCt of control), ΔCt treated= (Ct of target gene in treated group - Ct of 18 s rRNA gene in treated group) and ΔCt control= (Ct of target gene in control group - Ct of 18 s rRNA gene in control group [56].

**Table 2:** Primer sequence information of the genes involved in cordycepin biosynthesis.

Gene name	Gene symbol	Accession no.	Forward primer	Reverse primer
5′-nucleotidase 1	NT5E	KP090958	GCTCGTGTTCCTGCCATAGT	GCTCTACTATCGCCCGAGTG
Adenosine kinase	ADEK	KP090962	CCATTGGGCTCACGAGTCTT	CGTGACGAAAAGACAGTGCC
AMP deaminase	AMPD	KP090965	CCGTGAGCATGTCGAGGAG	GCAGCAAAACACCATCCGTC
Ribonucleoside-diphosphate reductase L subunit	RNR	KY435930	CAGTCCCAGTCGCTCAACAT	TCCTTCACATACGTGGCTCG
Phosphoribosyl-formyl-glycinamidine synthase	purl	KP090952	AGACCTTTGCGCTCGAGAAA	GGTACTGCATCTCGGACTCG
Adenylosuccinate synthase	purA	KP090957	CCGTACCCGAGACTTGACAC	AGGCAGTTGGGATTGACGAG
Adenylosuccinate lyase	purB	KP090955	CCTTTCCAAGTTTGCCTGCC	CGGCCCTAACGTATTCGACA
Phosphoribosyl-aminoimidazole-succinocarboxamide synthase	purC	KP090954	ACATACATCCCGACGATGCC	TTGTCGAAGCTGTCCTGGTC
Ribose-phosphate pyrophosphokinase 1	PRPS	KP090946	CACGACTACGAGAACCCCAG	TCGGGAATGTGCTTCACGAT
18 s ribosomal RNA	18 s rRNA	MG642880	GACGCGTTCGGCACCTTA	TTCAGCCTTGCGACCATAC

### 2.8 Statistical analysis

All experiments were performed at least thrice and data were represented as average mean ± SEM. The statistical significance was calculated by one-way or two-way ANOVA to assess the difference between group and their respective controls followed by an appropriate test such as Tukey’s multiple comparison test using Graph Pad Prism version 6.05.

## 3 Results and discussion

### 3.1 Hypoxanthine and adenosine enhance cordycepin production

**Fig. 1:**
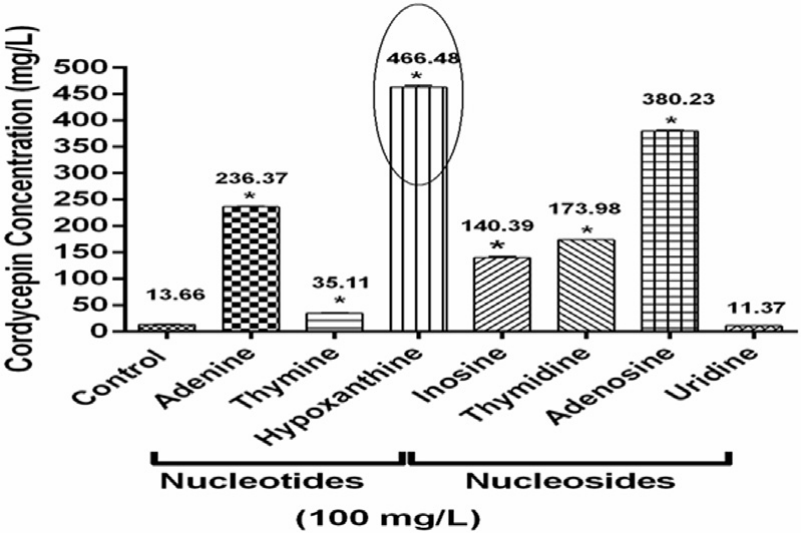
*Effect of nucleosides on cordycepin production in CS2973.* The graph representing the cordycepin concentration obtained after the addition of different nucleotides and nucleosides to the culture at 100 mg/L concentration. Data was presented as the mean ± SEM of 3 independent experiments. * indicating level of significance accepted at p ≤ 0.05 as compared to control.

Out of different nucleosides tested for cordycepin production, hypoxanthine depicted maximum amount of cordycepin production (466.48 ± 3.89 mg/L) followed by adenosine (380.23 ± 1.78 mg/L) whereas least amount of cordycepin was obtained with uridine (11.37 ± 0.23 mg/L) as compared to control (13.66 ± 0.64 mg/L) without any supplementation as shown in Fig. 1. However, the contribution of adenine, inosine, and thymidine cannot be neglected as they also induced cordycepin content up to 236.37 ± 1.17, 140.39 ± 2.34, and 173.98 ± 0.33 mg/L respectively. The increased production of cordycepin due to hypoxanthine and adenosine supplementation may be attributed to the fact that cordycepin is a nucleoside analog of adenosine, also adenosine is the main precursor for the biosynthesis of cordycepin which accelerated the cordycepin biosynthesis pathway [47]. Masuda et al. [42] also reported the enhanced cordycepin production using surface culture approach in *C. militaris* and it was revealed that adenine and adenosine significantly increased the cordycepin content as compared to control in the culture medium.

### 3.2 Supplementation of amino acids increases cordycepin production

**Fig. 2:**
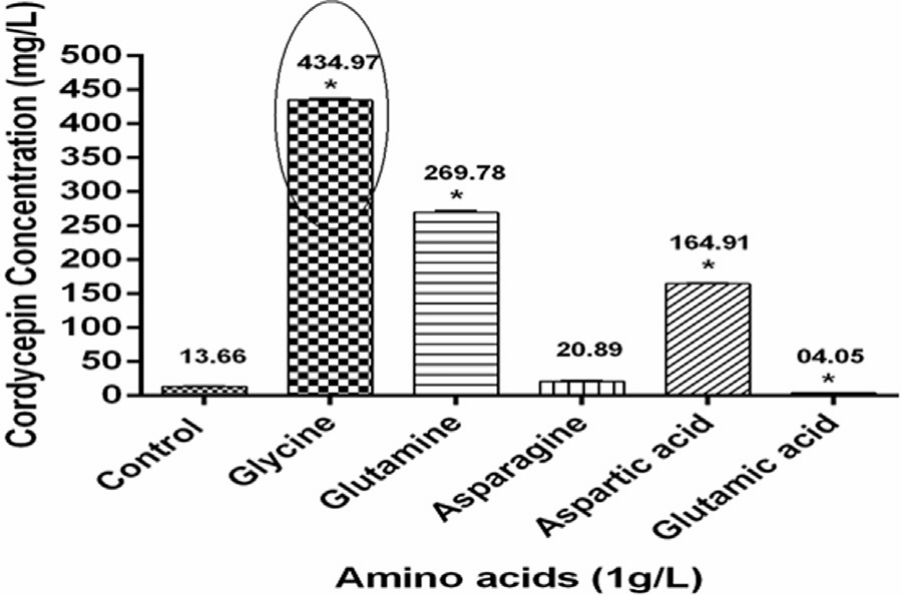
*Effect of amino acids on cordycepin biosynthesis in CS2973.*Graph depicting higher cordycepin production in mycelial biomass upon addition of glycine, glutamate, and aspartic acid as compared with control. Data was presented as the mean ± SEM of 3 independent experiments. * indicating significance level at p ≤ 0.05 as compared to control.

Since amino acids are known to be involved in cordycepin biosynthesis as the nitrogen source, this incited us to utilize amino acids for the enhancement of cordycepin production [48]. The maximum amount of cordycepin (434.97 ± 2.32 mg/L) was obtained when glycine was supplemented at 1 g/L concentration. Glutamine and aspartic acid also significantly increased the cordycepin content 269.78 ± 2.92 and 164.91 ± 1.19 mg/L respectively (Fig. 2). However, on glutamic acid supplementation in the culture medium, the cordycepin amount was reduced to 4.05 ± 0.42 mg/L as compared to control (13.66 ± 0.64 mg/L). Masuda et al. [42] reported that glycine and glutamine amino acids increased the cordycepin content 2.8 and 2.5 times higher respectively using the surface fermentation approach. However, the results depicted that using the submerged fermentation approach, cordycepin content increased 31.8 and 19.7 times using glycine and glutamine amino acids as compared to control. From these results, it can be concluded that glycine and glutamine are the major nitrogen sources required by CS2973 as compared to other amino acids which induced the cordycepin biosynthesis pathway.

### 3.3 Plant growth hormones- NAA and IAA potentiates cordycepin production

**Fig. 3:**
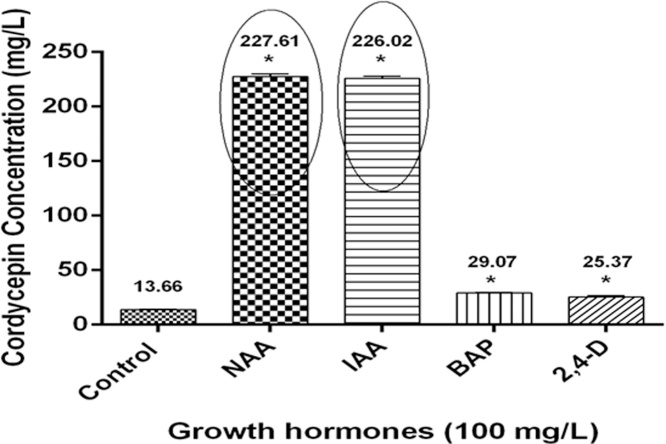
*Effect of plant growth hormones on cordycepin production in CS2973.* Cordycepin production was found to be higher in NAA and IAA at a concentration of 100 mg/L as compared to control. Data was presented as the mean ± SEM of 3 independent experiments. The significance level was accepted at p ≤ 0.05 as compared to control.

Plant growth hormones play a significant role in cordycepin production in *Cordyceps* spp. Some of these hormones are the precursors for enzyme synthesis involved in cordycepin biosynthesis [49]. Kang et al. [50] optimized cordycepin production in *C. militaris* and reported that NAA significantly increased the cordycepin production but utilization of plant growth hormones for cordycepin production in CS2973 has not been explored yet. When plant growth hormones were supplemented to the culture medium, it was observed that NAA and IAA significantly increased the cordycepin content up to 227.61 ± 2.34 and 226.02 ± 1.69 mg/L respectively as compared to control (13.66 ± 0.64 mg/L) as shown in Fig. 3. This increased cordycepin production in mycelial biomass suggested that plant growth hormones stimulate the production of cordycepin in CS2973 which can be utilized at an industrial scale.

### 3.4 Effect of vitamins on cordycepin production

**Fig. 4:**
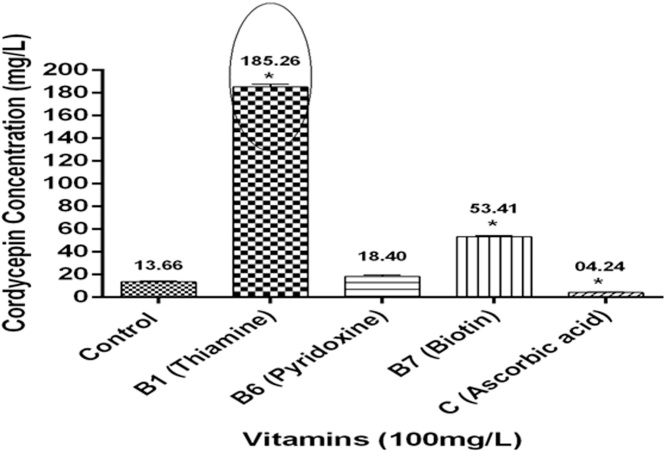
*Effect of different vitamins on cordycepin production in CS2973.*Graph representing higher cordycepin content in thiamine treated group as compared to control. Data was presented as the mean ± SEM of 3 independent experiments. The significance level was accepted at p ≤ 0.05 as compared to control.

Out of four types of vitamins tested, B_1_ was found to be the most appropriate vitamin for potentiating the cordycepin production as cordycepin amount raised to 185.26 ± 2.35 mg/L on supplementation at 100 mg/L concentration in liquid medium (Fig. 4). However, biotin also produced 53.41 ± 0.77 mg/L cordycepin content but surprisingly, vitamin C showed a significant reduction in cordycepin content 4.24 mg/L ± 0.31 as compared to control (13.66 ± 0.64 mg/L). Similarly, Kang et al. [50] also reported vitamin B_1_ as an active growth supplement in *C. militaris* for cordycepin production.

### 3.5 Differential mRNA expression analysis of genes involved in cordycepin biosynthesis

Several transcriptomic studies have investigated the genome of *O. sinensis* and *C. militaris* for identifying the genes involved in the cordycepin biosynthesis pathway to understand its downstream signaling molecular mechanism [2,13,46,57]. An array of genes have been identified in the literature to play a major role in cordycepin production such as *ADEK*, *NT5E*, *RNR*, *purA*, *purC*, *AMPD, etc* [2,57]. A cascade of enzymatic reactions takes place to synthesize cordycepin from its precursors. However, recent reports suggested *Cns1* and *Cns2* genes encoding for two crucial enzymes to be directly involved in the final steps of cordycepin biosynthesis in *C. militaris* at the transcriptional level [51,52]. Surprisingly, Xia et al. [52] reported that the *Cns1−4* genes are absent in *O. sinensis* suggesting the role of other unknown genes in this species for cordycepin production. The modulation of the genes or enzymes involved in cordycepin biosynthesis can enhance cordycepin production. For instance, ribonucleotide reductase small subunit, RNRM overexpression increases cordycepin production in *C. militaris* [46]. However, both RNR large and small subunits are essential for its activity and regulation of product specificity [53]. RNR, NT5E, and ADEK can be utilized as molecular targets to enhance cordycepin production in *O. sinensis* as these genes have a higher copy number in *Cordyceps* spp. [46,54]. Hence, in the present study, the growth supplements inducing these cordycepin synthesis related genes expression were considered potent enhancers of cordycepin production.

### 3.6 Nucleoside-mediated activation of genes related to cordycepin synthesis

**Fig. 5:**
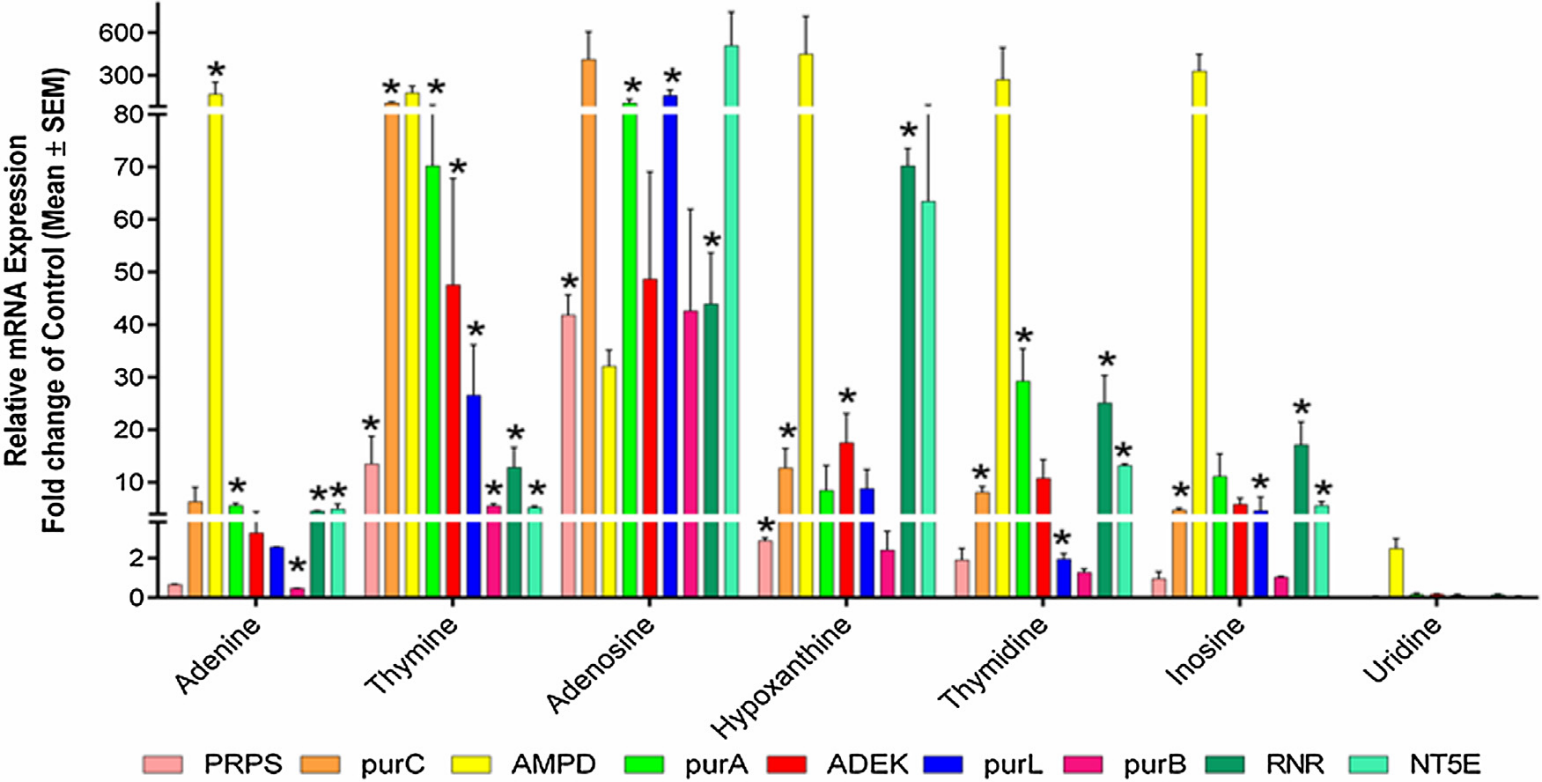
*qRT-PCR analysis to assess nucleotides and nucleosides mediated alteration in genes involved in cordycepin synthesis of CS2973.* Graph depicting mRNA expression profile of genes involved in cordycepin biosynthesis for group A (nucleotides and nucleosides) growth supplements. Data was presented as the mean ± SEM of 3 independent experiments. * indicating significance level was accepted at p ≤ 0.05 as compared to control.

To enhance the cordycepin biosynthesis, nucleosides were supplied to the culture medium. Interestingly, a significant upregulation of *RNR* (70.19 fold), *NT5E* (63.43 fold), *purA* (8.43 fold), and *ADEK* (17.52 fold) gene expression was observed upon hypoxanthine treatment which are downstream genes involved in cordycepin biosynthesis (Fig. 5). Though, hypoxanthine also induced the significant upregulation of other upstream genes of this pathway, suggesting its continuous requirement for their constitutive expression and importance during the cordycepin synthesis process. Being an analog of cordycepin, the effect of adenosine was also evaluated on cordycepin biosynthesis which also depicted a significant increase in mRNA expression of *RNR* (43.93 fold), *NT5E* (508 fold), *purA* (104.09 fold), and *ADEK* (48.7 fold) suggesting their essential role in cordycepin biosynthesis. Though there was a significant increase in *RNR* and *NT5E* due to other nucleosides also but it was not as high as hypoxanthine and adenosine. In agreement with RP-HPLC observations, uridine supplementation did not show any changes in the expression of any gene involved in the biosynthetic pathway.

### 3.7 Amino acids-mediated transcriptional changes in mRNA expression of genes responsible for cordycepin synthesis

**Fig. 6:**
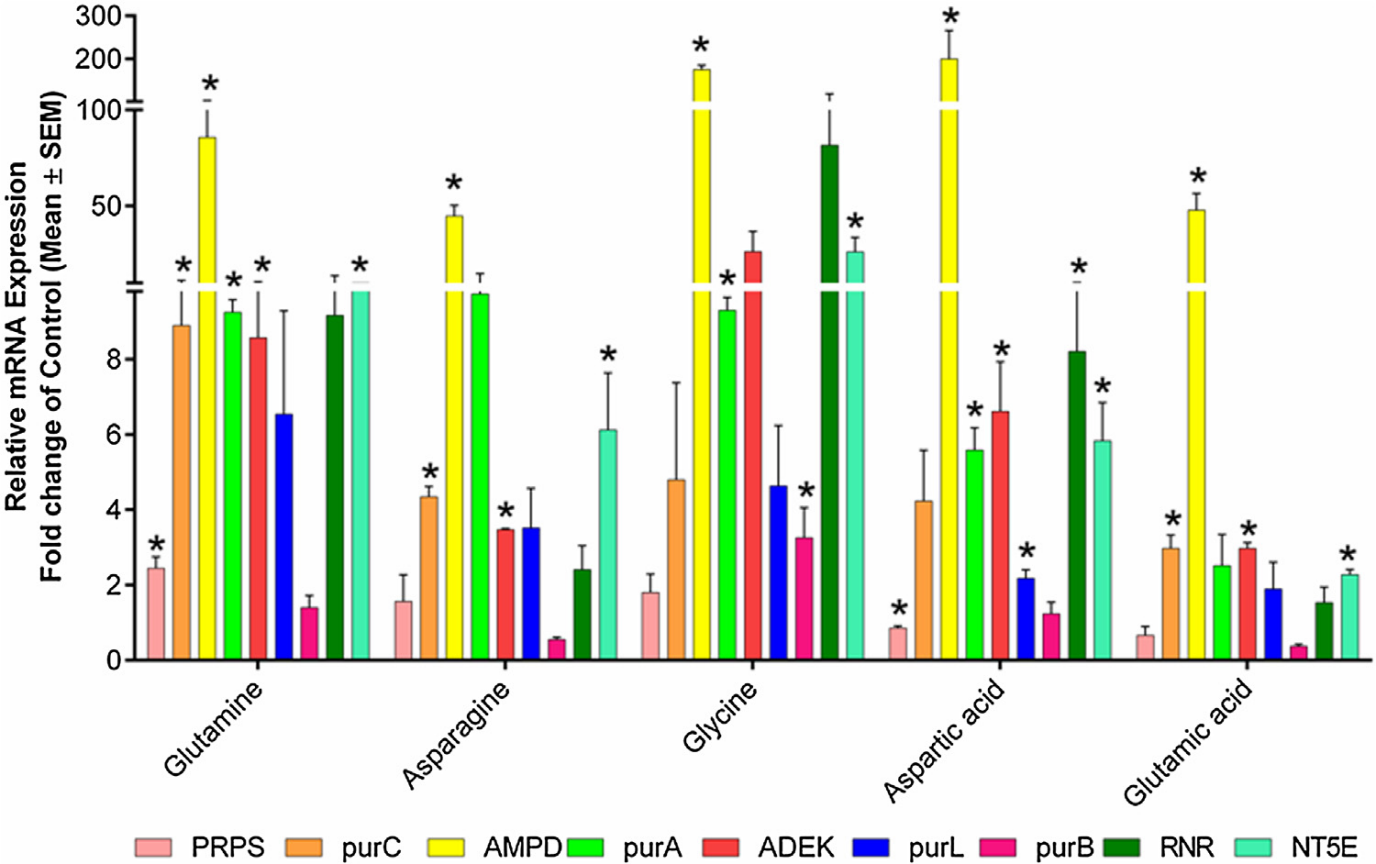
*Amino acids-mediated alterations at the transcriptional level.* Graph showing the mRNA expression pattern of genes involved in cordycepin biosynthesis for group B (amino acids) growth supplements. The major downstream genes *RNR* and *NT5E* were upregulated in glycine, glutamine, and aspartic acid. Data was presented as the mean ± SEM of 3 independent experiments. The significance level was accepted at p ≤ 0.05 as compared to control.

As depicted by RP-HPLC observations of amino acids supplementation in culture medium for cordycepin production, it was observed that glycine, glutamine, and aspartic acid significantly increased the cordycepin production (Fig. 2). To determine the gene expression profile of genes involved in cordycepin biosynthesis using amino acids as growth supplements, qRT-PCR analysis represented that glycine supplementation significantly upregulated the expression of *RNR* (81.68 fold), *NT5E* (26.20 fold), *purA* (9.30 fold) and *ADEK* (26.25 fold) genes. Glutamine and aspartic acid also showed the increased expression of *RNR* (9.17 and 8.20 fold), *NT5E* (9.95 and 5.83 fold), *purA* (9.25 and 5.59 fold), and *ADEK* (8.57 and 6.61 fold) genes as compared to control (Fig. 6). These findings suggested that glycine, glutamine, and aspartic acid are better growth supplements for enhancing cordycepin production as their supplementation significantly upregulated the expression of major gene targets (*ADEK, RNR,* and *NT5E*) of cordycepin biosynthesis.

### 3.8 Plant growth hormones induced differential expression of genes involved in cordycepin biosynthesis

**Fig. 7:**
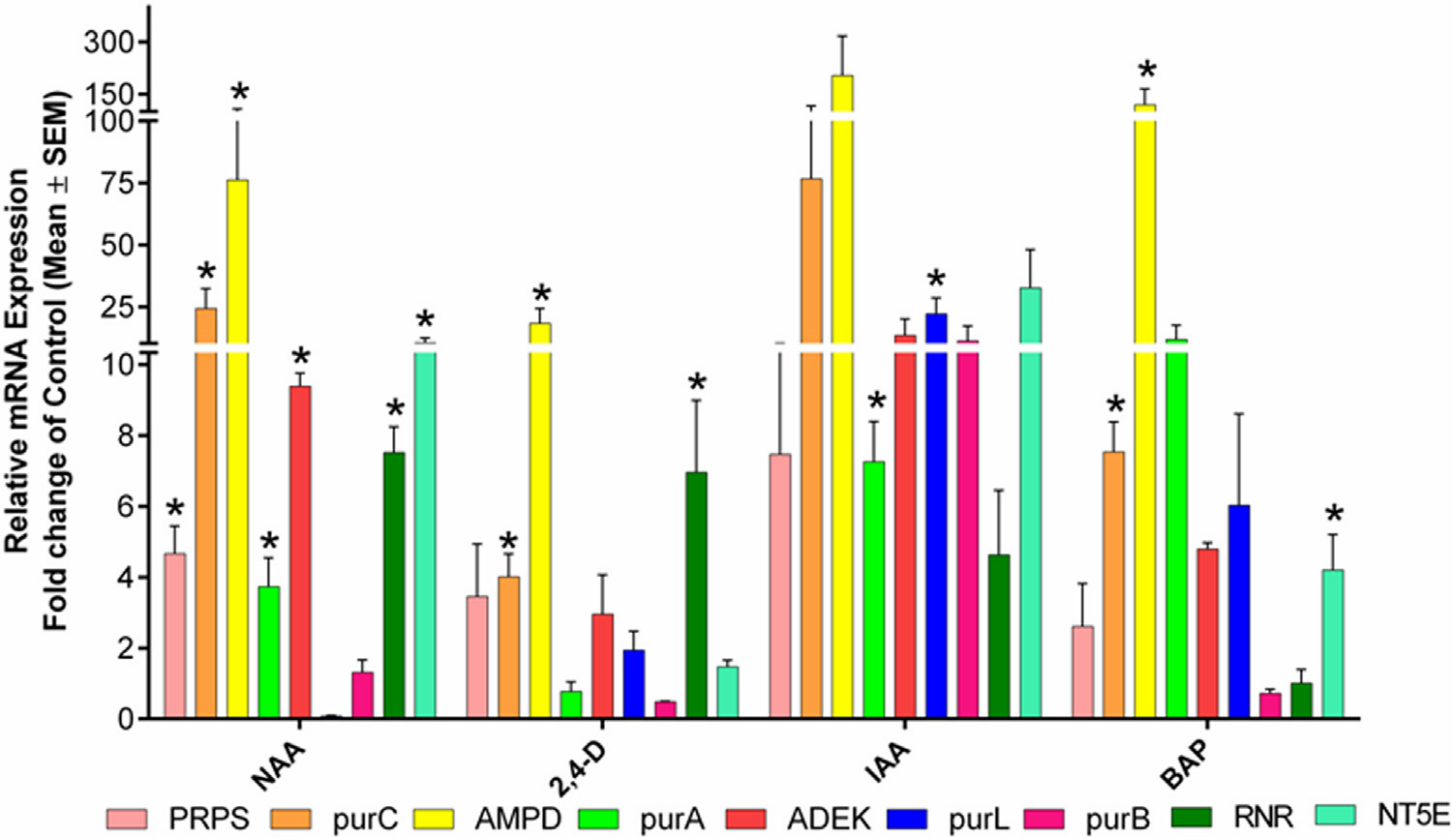
*Plant hormones-mediated changes in mRNA expression levels of genes responsible for cordycepin synthesis.* Graph representing the mRNA expression pattern of genes involved in cordycepin biosynthesis for group C (plant growth hormones) growth supplements. Data was presented as the mean ± SEM of 3 independent experiments. The significance level was accepted at p ≤ 0.05 as compared to control.

Plant growth hormones can be major inducers for cordycepin synthesis which has not been evaluated until now. The addition of plant growth hormones such as NAA, IAA, 2, 4-D, and BAP in culture medium showed differential expression of genes for cordycepin synthesis. NAA showed the upregulation of *RNR* (7.51 fold), *NT5E* (10.59 fold), *purA* (3.73 fold), and *ADEK* (9.38 fold) and IAA showed upregulation of *RNR* (4.63 fold), *NT5E* (32.79 fold), *purA* (7.25 fold) and *ADEK* (13.59 fold) genes as compared to control (Fig. 7). These results suggested that NAA and IAA act as potential growth supplements for increasing cordycepin production in CS2973 as their supplementation also enhanced cordycepin biosynthesis depicted by RP-HPLC.

### 3.9 Effect of vitamins on gene expression of genes involved in cordycepin biosynthesis

**Fig. 8:**
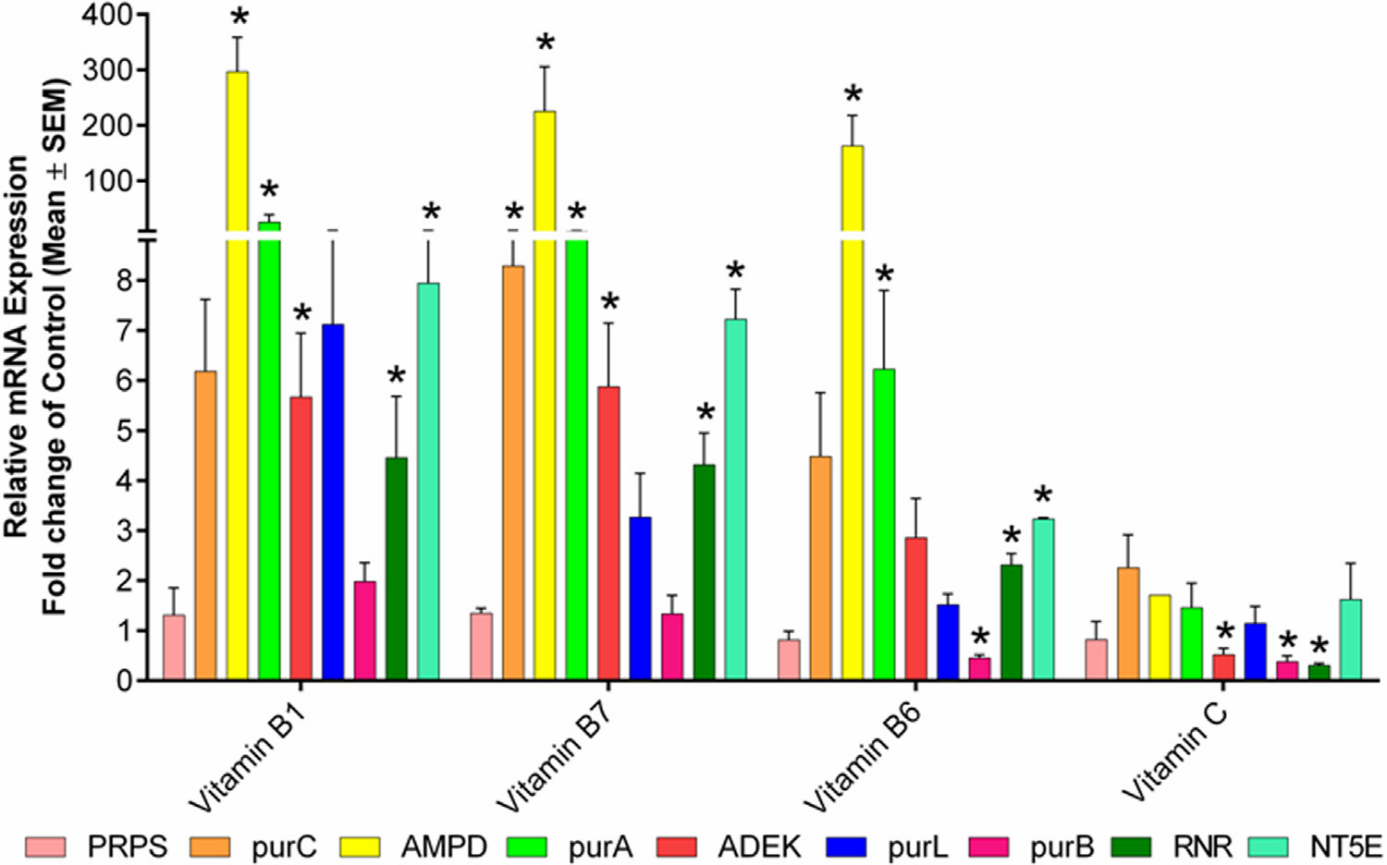
*Effect of vitamins on the mRNA expression profile of genes involved in cordycepin biosynthesis.* Among group D (vitamins) growth supplements, vitamin B_1_ and biotin could increase *RNR* and *NT5E* gene expression. Data was presented as the mean ± SEM of 3 independent experiments. The significance level was accepted at p ≤ 0.05 as compared to control.

Vitamins, specifically B-complex, play a significant role in the enhancement of cordycepin production in *Cordyceps* spp. [55]. This led us to evaluate the effect of vitamins on the transcription of genes participating in cordycepin production which has not yet been explored earlier. Four different types of vitamins (B_1_, B_6_, biotin, and C) were supplied in the culture medium to study their effect on cordycepin production. The results from the qRT-PCR analysis were corroborated well with the RP-HPLC analysis where B_1_ significantly increased the cordycepin content present in mycelial biomass of the CS2973. The gene expression profile upon treatment with B_1_ depicted a significant upregulation of *RNR* (4.46 fold), *NT5E* (7.94 fold), *purA* (25.37 fold), and *ADEK* (5.67 fold) genes (Fig. 8). So, B_1_ supplementation majorly increased the expression of *purA* gene which proved to be a major target for enhancing cordycepin biosynthesis in CS2973. However, Vitamin C did not induce the gene expression which is consistent with the RP-HPLC results suggesting its non-involvement in the pathway.

**Fig. 9:**
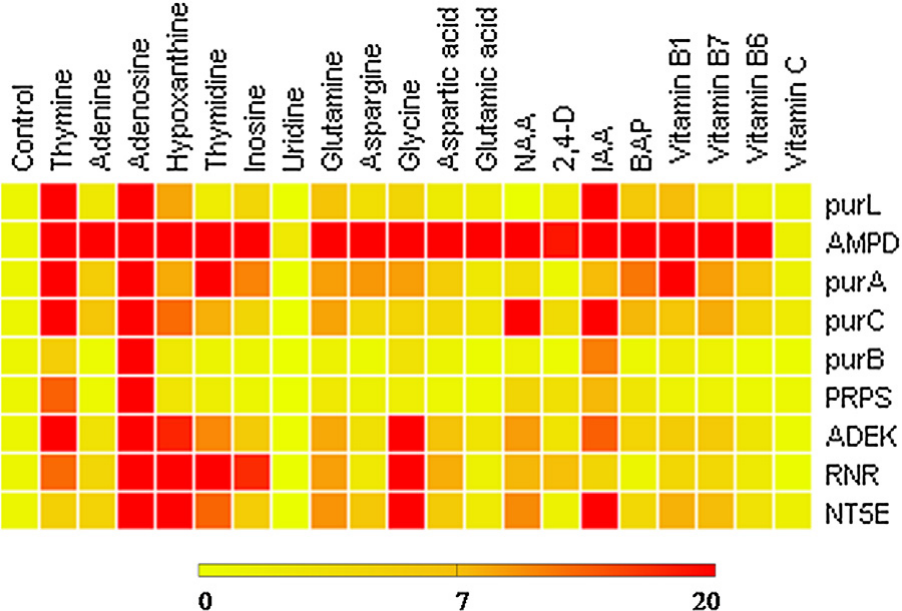
*Heatmap representing the differential expression of mRNA expression involved in cordycepin biosynthesis*: Although many additives activated the signaling mechanism by inducing expression of genes related to cordycepin biosynthesis, but hypoxanthine, adenosine, glycine, and IAA were observed to best growth supplements for enhancement of cordycepin production.

For further evaluation, a heatmap was generated depicting the differential regulation of genes playing role in cordycepin biosynthesis upon the addition of different growth supplements. The results depicted hypoxanthine, adenosine, glycine, IAA, and vitamin B_1_ serve as better supplements for the enhancement of cordycepin production in mycelial biomass of CS2973 (Fig. 9).

## 4 Conclusion

Cordycepin possessing a high therapeutic value and market demand have been extensively studied for its pharmacological effect as well as the mode of action mechanistically, but very little attention is paid to enhance the cordycepin biosynthesis. This study deals with the enhancement of bioactive metabolite, cordycepin production in *O. sinensis* using different growth supplements under submerged fermentation. The RP-HPLC analysis depicted hypoxanthine, adenosine, glycine, glutamine, aspartic acid, NAA, IAA, and thiamine (B_1_) a better growth supplement to enhance cordycepin production by enhancing *RNR*, *NT5E*, *ADEK,* and *purA* as compared with other supplements. The mRNA expression analysis provides a comprehensive overview of alterations in genes for cordycepin biosynthesis suggesting the targeted modulation of these genes will enhance cordycepin production. Taken together, this study not only enhanced the cordycepin production at a significant level which can aid in meeting the cordycepin market demand but also facilitates the propagation of this rare and endangered species as well as paves the way for unraveling the mechanism of cordycepin biosynthesis.

## CRediT authorship contribution statement

**Vikas Kaushik:** Conceptualization, Methodology, Data curation, Writing - original draft. **Amanvir Singh:** Methodology, Writing - review & editing. **Aditi Arya:** Supervision, Writing - review & editing. **Sangeeta Chahal Sindhu:** Supervision, Writing - review & editing. **Anil Sindhu:** Supervision, Conceptualization, Visualization, Writing - review & editing. **Ajay Singh:** Supervision, Writing - review & editing.

## Declaration of Competing Interest

The authors declare that they have no competing interests.
